# Risk Factors for Frequent Infections in Patients With Extremely Severe Motor and Intellectual Disabilities

**DOI:** 10.7759/cureus.36174

**Published:** 2023-03-15

**Authors:** Ryoko Kusaba, Keisuke Yasumasu, Kenichiro Konishi, Yasuhiko Yamato, Satoko Kikawa, Kenji Okumura, Junko Maeda, Shingo Morinaga, Makoto Matsukura, Akihiko Kimura

**Affiliations:** 1 Pediatrics, Kumamoto-Ashikita Medical Center, Kumamoto, JPN; 2 Pediatrics, Faculty of Life Sciences, Kumamoto University, Kumamoto, JPN; 3 Pediatric Surgery, The University of Tokyo, Tokyo, JPN

**Keywords:** frequent infection, hypertriglyceridemia, hypoalbuminemia, severe motor intellectual disabilities, bacterial infection

## Abstract

Background: At our institution, patients of all ages with extremely severe motor and intellectual disabilities (ESMID) receive comprehensive management similar to intensive care for “extremely ill patients.” Some patients with ESMID develop frequent infections that are difficult to manage. The purpose of this study was to identify risk factors for frequent infections in these patients.

Methods: Thirty-seven patients with ESMID who were treated for infections at our institution between September 2018 and August 2019 were retrospectively investigated. Frequent infection was defined as three or more episodes of infection and antimicrobial treatment in one year. Infection status and potential risk factors for frequent infections (patient background factors, severity score, hematological parameters, anthropometry index, and parenteral nutritional status) were examined in univariate and multivariate analyses.

Results: Frequent infections occurred in 11 of the 37 patients (29.7%) during the study period, including respiratory and urinary tract infections. Univariate and multivariate analyses suggested hypoalbuminemia (p<0.01) and hypertriglyceridemia (p<0.01) were independent risk factors for frequent infections.

Conclusions: Hypoalbuminemia and hypertriglyceridemia may be risk factors for frequent infections in patients with ESMID.

## Introduction

Severe motor disturbance with profound mental retardation is known as severe motor and intellectual disabilities (SMID). Patients with SMID have a variety of functional disorders and complications in multiple organ systems stemming from a central nervous system disorder and have an estimated intelligence quotient of <35, which is equivalent to a maximum developmental age of 24 months [1,2].

In Japan, it is estimated that there are approximately 47,000 patients with SMID, 30% of whom are treated in institutions. With advances in medical care, the number of new patients with SMID is increasing every year because newborns and children with severe diseases who were previously very difficult to save can now be saved. Patients with SIMD often require intensive medical care, and many patients have difficulty returning home. Meanwhile, the number of doctors and medical staff who provide treatment is limited, and the current situation in Japan is unique in that the same pediatricians and staff must be responsible for the care of patients with SMID from infancy to old age. There are various reasons for the limited ability of physicians in adult medicine to manage patients with SMID, including that physicians and nurses lack the necessary knowledge and skills, and that they lack experience in dealing with parents of SMID patients. SMID includes not only common neurometabolic diseases that develop in adulthood but also a number of congenital disorders, including neurological disorders, that are not familiar to physicians in adult medicine. In addition, patients with SMID are prone to severe infections and other diseases, and the situation becomes more complex with age with the emergence of drug-resistant organisms. As a result, patients and their parents may be resistant and distrustful of physicians who usually treat adults, and healthcare professionals may often avoid such situations. Many neurological diseases in patients with SMID are problematic for both adult physicians and pediatricians. While knowledge of the treatment and management of SMID has improved recently, the paucity of literature on this subject further contributes to difficulties in the treatment and management of these patients. In this study, we focused on infections as a major problem in the management of patients with SMID and aimed to explore predictive factors for recurrent infections in these patients.

At our institution, Kumamoto Ashikita Medical Center, patients with SMID who require continuous intensive care, mainly ventilator management, are managed in the " extremely severe patient unit” as patients with "extremely severe motor and intellectual disabilities" (ESMID). The specific criteria used in Japan are based on the Japanese Society for Rehabilitation of Persons with Disabilities (JSRPD). Patients with SMID are scored based on items such as motor function and ventilator management, and those with more than a certain number of points are designated as having ESMID. These patients require continuous intensive medical care centered on ventilator management, which is very labor-intensive due to the need for ongoing monitoring and detailed observation. Moreover, these patients often have difficulty communicating, so detection and diagnosis of complications are often delayed. Bacterial infections in particular are not only difficult to diagnose and treat in these patients but also recur frequently, tend to be severe, and are a leading cause of death [3-6]. Severe infections are a problem, of course, but even common infections such as pneumonia and urinary tract infections can become far more severe than expected in patients with ESMID. Frequent recurrent infections are a major problem for doctors, nurses, and other medical staff in daily practice when caring for these patients, as they require more intensive care than usual to treat infections. Therefore, the diagnosis and treatment of bacterial infections are important aspects of medical care for patients with ESMID, and furthermore, the prevention of these infections is a critical issue. The purpose of this study was to clarify the risk factors for frequent infection in patients with ESMID managed in an extremely severe patient unit.

## Materials and methods

Patients, definitions, and criteria

This retrospective observational study was performed in September 2019. ESMID patients were defined according to the JSRPD criteria used in Japan (Table 1) [7]. These criteria are based on the following: (1) motor function up to the sitting position; (2) ventilator management, dietary function, presence or absence of gastroesophageal reflux, and supplementary items (such as position change, regular catheterization, stoma); and (3) a total score for each item of ≥25 points that continues for at least 6 months. Patients with ESMID, from children to the elderly, require a considerable amount of routine care and close monitoring, and at our institution are managed in an extremely severe patient unit, which is similar to an intensive care unit. All patients require intensive care, including respiratory management, oxygen administration, enteral feeding and/or frequent suctioning, etc. Bacterial infections that occurred between September 2018 and August 2019 were reviewed. Information on the presence of frequent infections and potential risk factors was collected from the inpatient medical records. Frequent infections were defined as bacterial infections for which antimicrobial therapy were administered at least 3 times in 1 year [8]. Antimicrobial therapy could include either oral or intravenous administration and was continued until resolution of the patient’s infection was confirmed. Different doctors were involved in the patients’ care because they were managed in an intensive care unit where the doctors work in shifts. However, each patient has their own attending physician, and the treatment plan is decided through discussion among all the treating physicians. The patients were divided into a group with frequent infections (frequent group) and a group without frequent infections (non-frequent group). The following potential risk factors were compared between the two groups.

**Table 1:** Judgment criteria for patients with extremely severe motor and intellectual disabilities and patients with semi-super severe motor and intellectual disabilities (Japanese Society for Rehabilitation of Persons with Disabilities) If the motor function in item 1 is up to the sitting position and the total score in 2 is 25 points or more, the patient is considered to have extremely severe motor and intellectual disabilities, and if the score is 10 points or more but less than 25 points, the patient is considered to have semi-super SMID. If the condition specified in each of these items persists for six months or more, the respective scores are summed. For children who have been transferred from the neonatal intensive care unit, ongoing conditions must persist for one month or more. However, in the case of symptom exacerbation or the development of a new disease after leaving the neonatal intensive care unit, the condition must continue for six months or more. *1 Cuff machines, such as noninvasive positive-pressure ventilation and continuous positive airway pressure that require daily mechanical airway pressure are included in respiratory management. *2 For (8) and (9), choose between oral intake, enteral tube, and enteral tube feeding *3 Includes artificial bladder

1	Motor function: Up to sitting position	Yes/No
2	Judgment score (points)	Score
(1)	Respiratory management *1	10
(2)	Endotracheal intubation/tracheostomy	8
(3)	Nasopharyngeal airway	5
(4)	Oxygen administration or SaO_2_ below 90% for more than 10% of the time	5
(5)	Frequent suctioning more than once/hour	8
	Frequent inhalation more than 6 times/day	3
(6)	Nebulizer more than 6 times/day or continuous use	3
(7)	IVH	10
(8)	Oral intake (with full assistance) *2	3
	Tube (including nasal and gastric) *2	5
(9)	Enteral tube feeding	8
	Continuous infusion pump (during enteral tube feeding)	3
(10)	Overstrain that does not improve with surgery or medication, and changing clothes and correcting posture due to sweating more than 3 times/day	3
(11)	Continuous dialysis (including peritoneal perfusion)	10
(12)	Routine urinary drainage (more than 3 times/day) *3	5
(13)	Artificial anus	5
(14)	Position change 6 times/day or more	3

Clinical and laboratory findings

Patient background characteristics (age, duration of hospitalization, sex), severity score, hematological parameters (lymphocytes, total protein, albumin, triglycerides [TG], total cholesterol, alanine aminotransferase, alkaline phosphatase, creatine kinase, blood urea nitrogen, creatinine), anthropometric indices (weight-to-height ratio), and parenteral nutritional status (nutritional adequacy of tube feeding, energy intake, and water intake) were examined as potential risk factors. All assessments were made when the patient's condition was stable.

Statistical analysis

Continuous variables are shown as the median (range) and categorical variables as the number. Items found to have a two-sided p-value of <0.1 in univariate analyses (chi-square tests and Mann-Whitney U test) were entered into a logistic regression model for stepwise multivariate regression analysis. Variables with a two-sided p-value of <0.05 in multivariate analysis were considered statistically significant, and their diagnostic accuracy was evaluated by receiver-operating characteristic (ROC) curve analysis. Additionally, the chi-square test was used to calculate odds ratios and p-values based on the optimal cut-off values. JMP version 13 (SAS Institute Inc., Cary, NC) was used to perform all statistical analyses and to produce the figure.

## Results

Patient characteristics

Data for all 37 patients with ESMID (25 male, 12 female) admitted to the extremely severe patient unit during the study period were analyzed. The mean age was 28 years; three cases were aged 1-5 years, nine were aged 6-14 years, five were aged 15-19 years, and 20 were aged 20 years or older. The primary conditions were cerebral palsy, severe neonatal asphyxia, hypoxic-ischemic encephalopathy, spina bifida, sequelae of meningitis or traumatic subarachnoid hemorrhage, total anterior encephalocele, lissencephaly, spinal muscular atrophy type I, myotonic dystrophy, nemaline myopathy, and congenital cytomegalovirus infection. Eleven of the 37 patients (29.7%) had frequent infections, of whom nine had respiratory tract infections and two had urinary tract infections. The median number of infections was 10 (range, 3-56) (Table 2). The patients with very frequent infections were relatively young and a high proportion required ventilator management.

**Table 2:** Patient characteristics Alb, albumin; TG, triglycerides

Patient	Age, years	Sex	Primary disease	Frequency of infections in 1 year, n	Type of infection	Severity score	Ventilator	Tracheostomy	Diversion	Bladder catheterization	Alb, g/dL	TG, mg/dl
1	10	Female	Hypoxic-ischemic encephalopathy	12	Respiratory tract	39	+	+	-	-	3.4	580
2	12	Male	Hydranencephaly, cerebral palsy	3	Respiratory tract	37	+	-	+	-	3.3	49
3	13	Male	Sequelae of traumatic subarachnoid hemorrhage	3	Respiratory tract	34	+	+	-	-	3.8	501
4	13	Female	Congenital cytomegalovirus infection	13	Respiratory tract	37	+	+	-	-	2.5	127
5	14	Male	Sequelae of non-traumatic intracranial hemorrhage	4	Respiratory tract	27	-	+	-	-	3.6	62
6	15	Male	Hypoxic-ischemic encephalopathy	9	Respiratory tract	42	+	-	+	+	3	286
7	18	Male	Prader-Willi syndrome	10	Urinary tract	34	+	-	+	-	3.1	95
8	18	Male	Hypoxic-ischemic encephalopathy	30	Urinary tract	29	+	-	+	-	4.1	506
9	20	Female	Holoprosencephaly	8	Respiratory tract	39	+	+	-	-	3.2	354
10	54	Male	Lissencephaly	10	Respiratory tract	37	+	-	+	-	3.1	127
11	56	Female	Neonatal asphyxia, cerebral palsy	56	Respiratory tract	34	+	-	+	-	1.9	89

Univariate analysis

Table 3 compares the outcomes between the frequent group and the non-frequent group. Univariate analyses revealed that duration of hospitalization (p=0.07), hypoalbuminemia (p<0.05) and hypertriglyceridemia (p<0.05) were correlated with frequent infections. The median albumin level was 3.2 g/dL (range, 1.9-4.1) in the frequent group and 3.5 g/dL (range, 2.7-4.7) in the non-frequent group; the respective median TG levels were 127 mg/dL (range, 49-580) and 72.5 mg/dL (range, 41-39). There was no significant difference in mean severity score between the frequent group and the non-frequent group (37 points [range, 27-42] vs 34 points [range, 26-44]; p=0.36). Furthermore, there was no significant difference in anthropometric indices or nutritional status (Table 3).

**Table 3:** Univariate analysis of patients with frequent infection * Data are expressed as the median (range) or number (%). Alb, albumin; ALP, alkaline phosphatase; ALT, alanine aminotransferase; BUN, blood urea nitrogen; CK, creatine kinase; Cre, creatinine; T-chol, total cholesterol; TG, triglycerides; TP, total protein; WtHR, weight-to-height ratio

		Frequent group (n=11)	Non-frequent group (n=26)	P-value
Patient characteristics	Age, years	13.6 (8.3–54.5)	31.8 (3.7–64.6)	0.17
	Duration of hospitalization, years	6.5 (1–49.5)	22.4 (2.7–51.6)	0.07
	Sex (male), n (%)	7 (63.6%)	18 (69.2%)	0.74
	Severity score, points	37 (27–42)	34 (26–44)	0.36
	Ventilator support, n (%)	10 (90.9%)	19 (73.1%)	0.23
	Oxygen therapy, n (%)	8 (72.7%)	12 (46.2%)	0.14
	Endotracheal suction (>1 time/h), n (%)	4 (36.4%)	15 (57.7%)	0.24
	Tracheostomy, n (%)	5 (45.5%)	9 (34.6%)	0.53
	Tracheal separation, n (%)	6 (54.6%)	16 (61.5%)	0.69
Blood test	Lymphocytes	33.9 (15.3–46.4)	36.4 (15.9–66.9)	0.47
	TP, g/dL	6.6 (5–7–8.6)	7.0 (5.9–7.6)	0.29
	Alb, g/dL	3.2 (1.9–4.1)	3.5 (2.7–4.7)	<0.05
	TG, mg/dL	127 (49–580)	72.5 (41–398)	<0.05
	T-chol, mg/dL	186 (88–378)	163 (123–238)	0.30
	ALT, IU/L	43 (3–205)	24.5 (7–138)	0.11
	ALP, IU/L	552 (230–1257)	373.5 (99–888)	0.10
	CK, IU/L	21 (6–74)	28 (8–516)	0.17
	BUN, mg/dL	10.1 (3.9–18.3)	10.7 (3.1–39.7)	0.80
	Cre, mg/dL	0.15 (0.08–0.73)	0.195 (0.07–1.1)	0.25
Anthropometric indices	WtHR	2158 (1216–2709)	2175 (927–3022)	0.63
Nutritional status	Nutritional adequacy of tube feeding, %	46.3(40.5–117.3)	78.8 (24.4–175.3)	0.66
	Energy intake, kcal/kg	19.9 (9.0–64.9)	24.6 (4.9–83.3)	0.39
	Water intake, mL/kg	40.4 (20.1–58.7)	38.7 (14.4–111.1)	0.76

Multivariate analysis

Table 4 shows the results of the multivariable logistic regression analysis for frequent infections. Duration of hospitalization, hypoalbuminemia, and hypertriglyceridemia were identified as potentially significant risk factors in univariate analyses (both p<0.1). In multivariate analysis, hypoalbuminemia and hypertriglyceridemia were associated with a significantly greater risk of frequent infections (hypoalbuminemia, odds ratio 1.43, 95% confidence interval [CI] 1.07-1.8, p<0.01; hypertriglyceridemia, odds ratio 1.10, 95% CI 0.85-0.98, p<0.01).

**Table 4:** Multivariable logistic regression analysis of factors associated with frequent bacterial infection Alb, albumin; TG, triglycerides

	Frequent bacterial infection		
	Odds ratio	95% confidence interval	p-value
Alb	1.28	1.02–1.59	<0.01
TG	1.11	0.84–0.97	<0.01

ROC curve analysis

**Figure 1:**
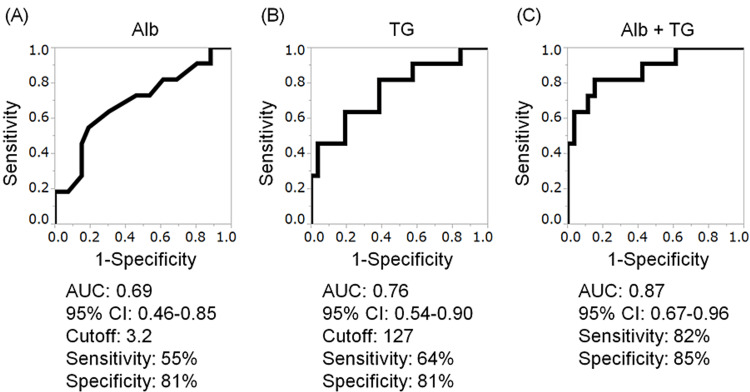
Receiver-operating characteristic (ROC) curves with cut-off values for hypoalbuminemia and hypertriglyceridemia alone and combined (A) Hypertriglyceridemia, (B) the combination of both hypoalbuminemia and hypertriglyceridemia (C) were obtained by plotting sensitivity against 1 - specificity. ROC curves for hypoalbuminemia. The AUCs are shown with the 95% confidence intervals. Alb, albumin; AUC, area under the ROC curve; CI, confidence interval; TG, triglycerides

ROC curve analysis was performed to identify the cut-off values for hypoalbuminemia, hypertriglyceridemia, and both hypoalbuminemia and hypertriglyceridemia for predicting frequent infections. The area under the ROC curve was 0.69 (95% CI 0.46-0.85) for hypoalbuminemia, 0.76 (95% CI 0.54-0.90) for hypertriglyceridemia, and 0.87 (95% CI 0.67-0.96) for the combination of hypoalbuminemia and hypertriglyceridemia. The combination of hypoalbuminemia and hypertriglyceridemia had sensitivity and specificity of 82% and 85%, respectively (Figures 1A-1C). In addition, the odds ratio with the optimal cut-off value for the combination of hypoalbuminemia and hypertriglyceridemia was 14.29 (95%CI 1.37-149.21, p<0.01).

## Discussion

In this study, 29.7% of patients with ESMID in an extremely severe patient unit were found to have frequent infections. Hypoalbuminemia and hypertriglyceridemia were suggested as potential independent risk factors in univariate and multivariate analyses. The treatment of children with SMID and their life expectancy have improved dramatically [9]. Treatment of patients with SMID requires input from both pediatricians and physicians in adult medicine. Ideally, treatment should be tailored according to age group, but this is not the case in Japan, where the management of patients with SMID is the responsibility of pediatricians, regardless of patient age. The reasons why physicians in adult medicine are unable to treat SMID include that physicians and nurses lack the necessary knowledge and skills, and that they lack experience in dealing with parents. SMID encompasses only common neurometabolic diseases that appear in adulthood such as adrenoleukodystrophy, but also many neurological diseases that develop in childhood and are not commonly encountered by physicians in adult medicine, including cerebral palsy, congenital cytomegalovirus infection, and sequelae of traumatic subarachnoid hemorrhage. Furthermore, patients with SMID are more likely to develop severe infections and other diseases, and with the emergence of drug-resistant bacteria, the situation becomes more complicated with age. As a result of these circumstances, both patients and parents can feel resistance and distrust toward physicians in adult medicine, and healthcare providers often avoid such situations. Therefore, we planned this study based on the situation in Japan, where pediatricians play a central role in the management of patients with SMID. This study is one of the first in ESMID to include patients with such a wide age range from childhood to old age. Various doctors diagnosed and treated the infections in this study. However, each patient had their own attending physician, and the treatment plan was decided through discussion among all the treating physicians. Therefore, we do not believe that treatment differences greatly from patient to patient. We consider that bias was reduced by having a variety of both doctors and patients involved in the study.

We found that hypoalbuminemia was a potential independent risk factor for frequent infections of ESMID. Hypoalbuminemia is common in SMID and has various causes, including poor nutrition, decreased synthesis of albumin in the liver as a result of oral medications that affect liver metabolism, and excessive consumption of albumin due to acute or chronic inflammation. Albumin is the most abundant plasma protein associated with oxidative stress and plays an important role in maintaining blood colloid osmolarity, metabolite transport, and nutrition in the body; thus, it can reflect the nutritional status of patients [10,11]. Patients with SMID often require multiple oral medications, including antiepileptic agents that are metabolized in the liver and cause hypoalbuminemia. Previous reports indicate that 46%-90% of patients with SMID are malnourished, and potential causes include severe neuropathy, gastroesophageal reflux, and constipation [12,13]. Nutritional management is often difficult in patients with SMID, and nutritional status is particularly poor in patients with ESMID. There is believed to be a link between malnutrition and inflammation, and hypoalbuminemia has been noted as an inflammatory marker associated with increased risk of poor clinical outcome [14]. Hypoalbuminemia can lead to weakened immunity and increased susceptibility to infectious diseases, and patients with sepsis have complex pathophysiological conditions, including immune imbalance and disorders of biosynthesis and catabolism [15]. Furthermore, hypoalbuminemia was found to be an independent predictor of fatal sepsis in an intensive care unit, with a 75.2% increase in the mortality rate among patients with serum albumin <2.9 g/dL [16]. Serum albumin levels are associated with the prognosis of pneumonia, severe sepsis, and bacteremia [17,18]. A finding of hypoalbuminemia always warrants investigation of multiple factors and active intervention.

This study also identified hypertriglyceridemia as a potential independent risk factor for frequent infections in patients with ESMID. Hypertriglyceridemia is associated with episodes of inflammation and infection [19]; the mechanisms are thought to include increased lipolysis and synthesis of TG during infection, which in turn can lead to severe pathology [20]. It has been found that catecholamines, lipopolysaccharide, and proinflammatory cytokines, including tumor necrosis factor-alpha, tumor necrosis factor-beta, interleukins 1 and 6, and interferon-alpha, are elevated in systemic inflammatory response syndrome (SIRS) and sepsis, rapidly leading to lipolysis of peripheral and visceral adipose tissue and release of free fatty acids (FFA) [19,21]. Production of a number of proteins, catecholamines, and cytokines, as well as lipopolysaccharide, accelerates utilization of FFA and synthesis of TG in the liver. Moreover, the clearance of TG, including its tissue availability, and oxidation of FFA are mediated by the action of protein lipase (PL). High levels of lipopolysaccharide inhibit PL activity, resulting in reduced TG clearance [19,22-24]. Therefore, in SIRS and sepsis, the main abnormality in the lipid profile is hypertriglyceridemia, which would also likely be found in patients with frequent infectious diseases.

After hypoalbuminemia and hypertriglyceridemia were identified as independent risk factors, ROC curve analysis was performed to assess their cut-off values for predicting frequent infections. The optimal cutoff values were 3.2 g/dL for albumin and 127 mg/dL for TG, with low areas under the curve of 0.69 and 0.76, respectively; however, the area under the curve for the combination of hypoalbuminemia and hypertriglyceridemia was considerably higher at 0.87. Moreover, the combination of these two variables had relatively high sensitivity and specificity and thus might be a reasonable predictor of frequent infections in patients with ESMD.

This study has a number of limitations. First, it included a small number of patients, and the weaknesses of the statistical analysis are undeniable. Nevertheless, we believe that this report is valuable in light of the increasing longevity of patients with SMID in whom infections are common [9]. A prospective multicenter controlled study involving a larger population should provide further information, and this will be a focus of future work. Second, although hypoalbuminemia and hypertriglyceridemia were identified as independent risk factors for frequent infections, whether these abnormalities were the cause or result of these infections is unclear. However, in patients with SMID who develop more than three infections in one year, the risk of further infections is high without intervention. Therefore, we believe that hypoalbuminemia and hypertriglyceridemia can be regarded as risk factors for further infections in the future. Third, the retrospective design of the study meant that our ability to investigate multiple items was limited. Although we anticipated that hypertriglyceridemia would be a risk factor for frequent infections in patients with SMID and that lipid synthesis would be abnormal, we were unable to measure very low-, low-, or high-density lipoproteins, which would have allowed a more accurate evaluation of lipid synthesis. A prospective study is now needed to determine if treatment of hypoalbuminemia and hypertriglyceridemia can prevent frequent infections in patients with ESMID.

## Conclusions

This study identified hypoalbuminemia and hypertriglyceridemia as independent risk factors for infection in both children and adults with ESMID in the extremely severe patient unit. In Japan, there are not enough doctors specializing in patients with SMID, and in reality, there are many special situations in which pediatricians treat patients with SMID from infancy to old age, especially outside of major cities. Therefore, the management of these patients is often difficult. This is the first publication of from a facility for SMID patients like ours. Therefore, we believe it will be very useful for both pediatricians and physicians in environments where they have to treat patients with SMID, from childhood to old age. In the future, we plan to conduct a study with the participation of multiple facilities and to analyze a larger number of patients.
